# Integrating support persons into maternity care and associations with quality of care: a postpartum survey of mothers and support persons in Kenya

**DOI:** 10.1186/s12884-024-06611-y

**Published:** 2024-06-13

**Authors:** Michelle K. Nakphong, Patience A. Afulani, Hiram Beltrán-Sánchez, James Opot, May Sudhinaraset

**Affiliations:** 1 Department of Medicine, Division of Prevention Science, University of California, San Francisco, CA USA; 2 Department of Epidemiology and Biostatistics, University of California, San Francisco, CA USA; 3 Department of Community Health Sciences, Fielding School of Public Health, University of California, Los Angeles, CA USA; 4 California Center for Population Research (CCPR), University of California, Los Angeles, CA USA; 5 Innovations for Poverty Action, Nairobi, Kenya; 6 550 16th St., 3rd Floor, San Francisco, CA 94158 USA

**Keywords:** Maternal health services, Social support, Quality indicators, Health care, Infant health, Communication

## Abstract

**Background:**

Despite research that has shown that the presence of support persons during maternity care is associated with more respectful care, support persons are frequently excluded due to facility practices or negative attitudes of providers. Little quantitative research has examined how integrating support persons in maternity care has implications for the quality of care received by women, a potential pathway for improving maternal and neonatal health outcomes. This study aimed to investigate how integrating support persons in maternity care is associated with multiple dimensions of the quality of maternity care.

**Methods:**

We used facility-based cross-sectional survey data from women (*n* = 1,138) who gave birth at six high-volume facilities in Nairobi and Kiambu counties in Kenya and their support persons (*n* = 606) present during the immediate postpartum period. Integration was measured by the Person-Centered Integration of Support Persons (PC-ISP) items. We investigated quality of care outcomes including person-centered care outcomes (i.e., *Person-Centered Maternity Care (PCMC)* and *Satisfaction with care*) and clinical outcomes (i.e., *Implementation of WHO-recommended clinical practices*). We used fractional regression with robust standard errors to estimate associations between PC-ISP and care outcomes.

**Results:**

Compared to low integration, high integration (≥four woman-reported PC-ISP experiences vs. <4) was associated with multiple dimensions of quality care: 3.71%-point (95% CI: 2.95%, 4.46%) higher PCMC scores, 2.76%-point higher (95% CI: 1.86%, 3.65%) satisfaction with care scores, and 4.43%-point (95% CI: 3.52%, 5.34%) higher key clinical practices, controlling for covariates. PC-ISP indicators related to communication with providers showed stronger associations with quality of care compared to other PC-ISP sub-constructs. Some support person-reported PC-ISP experiences were positively associated with women’s satisfaction and key practices.

**Conclusions:**

Integrating support persons, as key advocates for women, is important for respectful maternity care. Practices to better integrate support persons, especially improving communication between support persons with providers, can potentially improve the person-centered and clinical quality of maternity care in Kenya and other low-resource settings.

**Supplementary Information:**

The online version contains supplementary material available at 10.1186/s12884-024-06611-y.

## Background

Calls to improve the quality of maternal and newborn health care in Kenya highlight the need for preventive interventions during labor, delivery, and postpartum periods [1]. Poor quality of maternity care contributes to high rates of maternal and newborn mortality and morbidity; 90% of maternal deaths in Kenya are linked to substandard maternity care [2]. Women are also frequently mistreated by providers in maternity care. One-in-five Kenyan women in one study were treated with disrespect or felt humiliated during the childbirth experience [3]. 

Access to a support person during intrapartum care is one indicator of respectful, evidence-based care [4]. The World Health Organization (WHO) Quality of Care (QoC) Framework for Maternal and Newborn Health identifies social support as an essential component of quality care which, in turn, is posited to improve person-centered and clinical outcomes [5], but little quantitative evidence supports these proposed links. Recommendations to integrate support persons into maternity care highlight it as a low-cost, person-centered, and asset-based strategy to improve the quality care, but barriers to access needed social support remain [6]. We define a support person broadly, as a lay person (i.e., not a medical professional employed by the facility) who accompanies a woman to the facility or stays in or near the maternity ward during labor, delivery, or postpartum [7]. 

Support persons are vital advocates for women during maternity care, can influence providers interactions with women, and can increase person-centered care [8]. A study in Kenya found that women with family or friends present during maternity care were half as likely to report that health providers made them feel uncomfortable [3]. Another study in India found that women who had support persons included in discussions with providers were half as likely to report mistreatment [9]. A multi-country study also found that women with support persons were less likely to report physical abuse from providers and more likely to report that staff listened and responded to their concerns [10]. 

In Kenya and many global settings, provider and facility barriers to support persons persist in maternity care, such as prohibiting birth companions in maternity wards and negative treatment from providers [11, 12]. Some studies have shown that the proportion of women who were allowed a companion in Kenya is still low (only 6–42% and 4–16% of women report being allowed labor and delivery companionship, respectively) [12, 13]. Notably, even when support persons are allowed to be present during labor and delivery, they are not well integrated into care processes, hindering their support of women [14]. Most quantitative studies neglect to capture this lack of integration, only indicating birth companions’ presence without assessing the quality of engagement in care or ways that support persons’ involvement was facilitated, such as providing opportunities for women to consult support persons on decisions or facilitating communication between women support persons and providers [15]. Because engaging women, families, and communities in maternal and newborn health is essential for increasing patient safety, improving treatment and interactions with the health care system, we must identify practical strategies to better integrate support persons in maternity care [16]. 

We aimed to empirically investigate the mechanisms between integrating support persons and improved quality of care proposed in the WHO quality of care framework by testing associations between integrating support persons and quality of care outcomes. We operationalized integration of support persons by using the concept and measures of Person-centered Integration of Support Persons (PC-ISP) into maternity care, published and described in detail elsewhere [17]. PC-ISP builds on the concept of Person Centered Maternity Care which is defined as the delivery of care that is respectful of and responsive to women’s needs and preferences, ensuring that it guides all clinical decisions [18, 19]. We define PC-ISP as *the extent to which support persons are integrated into care* that is respectful and responsive to women’s needs and preferences, ensuring that women’s needs and preferences guide clinical decisions [19]. Based on themes in literature, PC-ISP highlights four specific domains that support persons can be integrated into maternity care: *Welcoming environment, Decision-making support, Communication and provision of information*, and *Ability to ask questions and express concerns*.

We grounded our study in the WHO Quality of Care Framework for Maternal and Newborn Health as part of the vision to make high-quality reproductive, maternal, and newborn health care available, accessible, and acceptable for all who need it [20]. The framework conceptualizes quality of care as comprising inter-linked dimensions of the provision and experience of care [5]. The provision of care refers to the clinical and technical delivery of care and the experience of care refers to how women were treated by providers and their perceptions of service, including whether they received adequate social support. Guided by the WHO QoC Framework for Maternal and Newborn Health, we hypothesized that integrating support persons is positively associated with people-centered outcomes (i.e., *person-centered maternity care*, *satisfaction with care*) and clinical outcomes (i.e., *implementation of key clinical practices*).

Following a person-centered approach, we sought to assess and compare women’s and support persons’ experiences. Examining the experience of care from women’s perspective is critical, asserting that women should be at the center of their own care [19]. A person-centered approach also recognizes that support persons are also participants and beneficiaries of health systems [21]. Moreover, the experiences and perspectives of support persons are under-studied and underrepresented.

## Methods

This study used women’s and support persons’ survey data from the Strengthening Person-centered, Accessibility, Respectful Care and Quality (SPARQ) study in Kenya. The SPARQ study worked with health facilities in Kenya to measure and improve person-centered care in maternity, family planning and abortion services. Women were surveyed at six high-volume (≥100 deliveries/month) urban public and private facilities (three public hospitals, two private hospitals, one public health centre) in Kiambu and Nairobi counties between September 2019–January 2020.

Women’s inclusion criteria included (a) aged 15–49 years; (b) speaking English or Kiswahili; (c) delivery via vaginal birth; and (d) owning a mobile phone and feeling comfortable to be contacted by the study team. Women were recruited from postpartum wards and research assistants obtained informed consent and administered face-to-face interviews using a structured questionnaire about their experiences of care in a private setting (~ 1 h), for instance an unused room or space within the facility or just outside the facility. Research assistants were trained to find a setting where privacy could be maintained throughout the visit including the introduction, consenting, and interviewing process. A total of 1,197 women were interviewed and the final analytic sample of women (*N* = 1,138) excluded 59 women without any reported support persons. Surveys asked respondents about demographics, health status, maternity care received, and their perceptions of care.

More than half of women (54%) identified one support person at the facility for potential participation in the support persons’ survey. Inclusion criteria for support person s included: (a) accompanied the woman to the hospital, stayed and assisted the woman during labor and/or delivery, or visited during the postpartum period; (b) ≥18 years; and (c) spoke English or Kiswahili. A total of 606 support persons were interviewed in a private setting for approximately 20 min.

Study procedures were approved by the Institutional Review Boards at the University of California, San Francisco (protocol number 19-27783) and the Kenya Medical Research Institute (Protocol KEMRI Non-SSC 666). Informed consent was obtained from all participants prior to participation.

## Measures

### Independent variables: person-centered integration of support persons (PC-ISP)

We used PC-ISP survey measures for women’s and support persons’ surveys which cover four sub-constructs: *Decision-making support, Communication and provision of information*, *Welcoming environment*, and *Ability to ask questions and express concerns* (see Table 1 for all measures) [17]. A *welcoming environment* highlights the importance of positive interpersonal relationships between providers and women’s preferred support persons [22, 23]. Adequate *decision-making support* promotes women’s autonomy and agency in their own care by providing opportunities to consult with support persons about clinical decisions [24]. *Communication and provision of information* facilitates support persons’ involvement in care and clarifies their roles [15, 25]. The *ability to ask questions and express concerns* provides opportunities for support persons to engage with providers during care, especially as an avenue to advocate on behalf of women [26]. 

Women’s PC-ISP measures (5-items) showed poor reliability (α = 0.592), indicating a fair amount of multidimensionality among the few indicators [27]. Response options (agree, somewhat agree, or disagree) were dichotomized (agree vs. disagree) because of the small fraction of “somewhat agree” responses (< 5% for all items). Correlations between individual PC-ISP indicators ranged from *r* = 0.037–0.62. We created a composite score of women’s PC-ISP measures (range 0–5), summing ‘agree/somewhat agree’ responses across indicators and dichotomizing based on the median: high PC-ISP (agree to 4 or more PC-ISP measures) vs. low PC-ISP (3 or less). For the score, we considered missing PC-ISP responses as equal to zero and conservatively recoded “Don’t know” responses as agree. Support persons’ PC-ISP measures (3-items) also displayed poor reliability (α = 0.594). Due to few support person PC-ISP measures, all support person-reported PC-ISP indicators were analyzed separately.

### Dependent variables: quality of care outcomes

Using a person-centered approach, we assessed outcomes by women’s self-report [23]. Following the WHO QoC Framework [5], we examined person-centered outcomes: *Person-Centered Maternity Care (PCMC)* and *Satisfaction with care;* and clinical outcomes: *Implementation of key practices.* All three outcome variables are continuous measures. The *PCMC* scale (long-version) measures women’s experience of care and has demonstrated high reliability and validity in Kenyan populations [18]. *Satisfaction with care* combined three items assessing satisfaction with: labor and delivery, after delivery, and newborn care. *Implementation of key practices* summed each woman’s reported receipt of 28 key practices from WHO’s standards of maternal and newborn care (detailed in Table 1) [28]. We also examined maternal and newborn key practices separately.

**Table 1:** Outcome and covariate measures

Domain	Variable name	Description of measure
***Person-centered integration of support persons (PC-ISP) measures (independent variables)***		
*Women’s PC-ISP variables*		
Decision-making support	*Opportunity to consult*	“I was given the opportunity by my health provider to consult my family about my health care decisions.”
Communication and provision of information	*Told condition/care*	“I was asked by my health provider if my family should be told about my condition and care.”
Welcoming environment	*Felt welcome*	“My family member(s) felt welcome by the facility at my delivery.”
Ability to ask questions and express concerns	*Welcome to ask questions*	“My family was welcome to ask my health care provider questions.”
	*Listened to concerns*	*“*My health care provider listened to my family members’ concerns.”
*Support persons’ PC-ISP variables*		
Communication and provision of information	*Provided info about woman*	“Were you provided resources or information from the mother’s health provider on how to help care for *the mother*?”
	*Provided info about newborn*	“Were you provided resources or information from the mother’s health provider on how to help care for *the newborn*?”
Ability to ask questions and express concerns	*Welcome to ask questions*	“Were you or do you think you would have been welcome to ask the health care providers questions about the mother and baby’s care?”
***Outcome measures (dependent variables)***		
*Person-centered outcomes*	*Person centered Maternity Care Scale (PCMC), long version*	The 30-item PCMC scale covers three subdomains: *Dignity and Respect* (6 items), *Communication and Autonomy* (9 items), and *Supportive Care* (15 items). Total and sub-domain scores were calculated by summing items and then standardized to 100-point scales for interpretability and comparability.
	*Satisfaction with care*	3-items assessing satisfaction with: labor and delivery, after delivery, and newborn care. Response options (Very satisfied; satisfied, dissatisfied, very dissatisfied) were summed and standardized to a 100-point scale.
Clinical outcomes	*Implementation of key practices*	Sum of reported receipt of 28 key practices from WHO’s standards of maternal and newborn care (range 0–28). Two sub-domains were analyzed including: *Maternal key practices* (17 items) included pre-delivery practices: whether a provider asked how she was feeling, if she was checked for headaches, bleeding, water breaking, had an examination, had blood pressure check, pulse check, contractions timed, fetal heartbeat assessed, a vaginal exam; and post-delivery practices such as if she was asked about pain, blood pressure check, pulse check, abdominal, perineum, and bleeding examinations, and whether staff were always accessible. *Newborn key practices* (11 items) included post-birth infant examination, put immediately on the mother’s chest after delivery, wiped dry, not bathed in the first 6 h, had temperature assessed, cord examined, and whether a health provider counseled on newborn danger signs, checked if breastfeeding was going well, observed breastfeeding, helped demonstrate breastfeeding, and if breastfeeding was initiated in the first hour after birth.
***Covariates (additional independent variables)***		
Women’s factors	Age, marital status, parity, educational attainment, current employment status, birthplace, health insurance coverage, and self-rated health status	
Support Person factors	Support person type(s), total support persons, and timing of support. Additional covariates for support persons’ PC-ISP analyses: support person occupation and accompaniment to antenatal care as a measure of prior support. *Facility factors* included facility type and number of providers assisting delivery. We also considered potential selection effects: whether women selected the facility because of quality and being referred to the facility.	
Household factors	Household empowerment: women who were married or partnered were asked decision-making power questions regarding woman’s health care, major household purchases, daily household purchases, and visits to family or relatives. Women who reported involvement in all four decisions (i.e., ‘woman only’ or ‘jointly’) and those not married/partnered were coded as *empowered in household decisions*.	
Provider/Facility factors	Facility type, number of providers assisting delivery, whether women selected the facility because of quality, and having been referred to the facility.	

### Analysis

We conducted descriptive analysis of the sample, PC-ISP variables, and outcomes using univariate and bivariate statistics. We performed two sets of analyses, separately examining 1) women’s PC-ISP and 2) support persons’ PC-ISP measures. For each set of analyses, we used fractional probit regression to estimate associations between PC-ISP and quality of care outcomes. Fractional regression is appropriate for modeling continuous dependent variables over a defined bounded range. For model estimation, we scaled dependent variables PCMC, satisfaction, and key practices to a 0–1 scale and obtained average marginal effects to estimate percent change in the dependent variables. For women’s PC-ISP, we examined models for the PC-ISP score (continuous and dichotomous variables) and individual PC-ISP indicators, excluding observations with missing PC-ISP data. For support persons’ PC-ISP, we examined individual PC-ISP indicators. We adjusted for covariates at multiple levels to address potential confounding including women’s, support persons’, household, and provider/facility factors (Table 2). To account for potential clustering by facility, we used robust standard errors. We checked normality of residuals for the continuous outcome variables in multivariate linear regressions and found that the residuals departed from normality for PCMC and Implementation of key practices, indicating that assumptions required for linear regression would be violated. Sensitivity analyses included fitting linear regression models, including facility fixed effects, random-intercept models by facility, constructing PC-ISP scores as a continuous variable and scores omitting items with low factor loadings, and using other functional forms such as negative binomial and log-transformation of outcomes because residuals were non-normal. However, estimates were consistent leading to similar substantive conclusions across sensitivity analyses. For example, comparing estimates from linear regression models to fractional regression results, fractional regression estimates were lower in magnitude but higher in statistical significance and similar in pattern. We also examined models dropping “don’t know” PC-ISP responses and found that coefficient estimates differed only by 0.1-5.4% and that including “don’t know” responses biased estimates to be slightly more conservative. Final fractional regression models excluding facility fixed effects presented in the results were the best fitting models according to AIC/BIC. We also examined potential synergistic effects between women’s and support persons’ PC-ISP measures using statistical interactions, but we found little evidence of interactions. We used STATA 15.1 (StataCorp LP, College Station, TX, USA) for all analyses and assumed alpha = 0.05 for statistical significance.

## Results

Women were, on average, 25.4 years old (SD 5.0), multiparous (61.8%), born outside of Nairobi or Kiambu (79.0%), and tended to attain primary or less education (44.3%) compared to vocational/secondary (39.9%) (Table 2). Women reported an average of 1.5 (SD 0.7) support persons including male partners (60.0%), sisters (16.7%), mothers (8.5%), other family members (21.2%), and friends/neighbors/others (34.7%). A small fraction reported a support person present with them during labor/delivery (7.4%) but almost all were accompanied to the facility (94.6%). Most women delivered at a government hospital (73.3%). The 606 support persons surveyed were male partners (42.7%), mothers/mothers-in-law (8.7%), other family members (28.5%), and friends/neighbors/other (20.0%). Many (42.9%) reported accompanying women to antenatal care appointments.

**Table 2:** Sample of women and support persons

Sample of women	*N* or mean	% or (SD)	
***Total women surveyed***	1,138		
Age			
Mean age	25.4	(5.0)	
Parity			
Mean parity	2.0	(1.0)	
Primiparous	435	38.2%	
Married/partnered	944	83.0%	
Educational attainment			
Primary or less	504	44.3%	
Vocational/Secondary	454	39.9%	
College/University	180	15.8%	
Currently employed	451	39.6%	
Birthplace			
Born in Nairobi or Kiambu counties	239	21.0%	
Born elsewhere	899	79.0%	
Self-rated health status			
Excellent or very good	398	35.0%	
Good	456	40.1%	
Fair	181	15.9%	
Poor or very poor	103	9.1%	
Support person types reported by women*			
Male Partner	683	60.0%	
Mother/mother-in-law	129	11.3%	
Other family member	424	37.3%	
Friend/neighbor/other	395	34.7%	
Total number of support persons			
Mean (min 1- max 6)	1.5	(0.7)	
Timing of support from any support person*			
Had support person(s) accompany to facility	1,076	94.6%	
Had support person(s) present during labor and/or delivery	84	7.4%	
Had support person(s) present postpartum	497	43.7%	
Empowered in household decisions			
Not involved in all decisions	518	45.5%	
Involved in all decisions	620	54.5%	
Facility type			
Gov’t hospital	834	73.3%	
Gov’t Health Centre/Dispensary	137	12.0%	
Private facility	167	14.7%	
Providers assisting delivery	1.1	(0.4)	
Selected facility because of quality	733	64.4%	
Referred to facility	180	15.8%	
**Sample of Support Persons**	**N or mean**	**% or (SD)**	
***Total Support Persons surveyed***	606		
Support person’s relationship to woman			
Male partner	259	42.7%	
Mother/Mother-in-law	53	8.7%	
Other Family	173	28.5%	
Friend/Neighbor/Other	121	20.0%	
Support person’s occupation			
Casual labor	125	20.6%	
Self-employed in petty trade or small-scale industry	220	36.2%	
Salaried worker	128	21.1%	
Unemployed/homemaker	135	22.2%	
Support person accompanied woman to any antenatal care	260	42.9%	

* Percentages do not sum to 100% because women could mark multiple response options (i.e., report multiple support persons, timings of support)

Women most frequently reported that providers listened to support persons’ concerns (82.0%) and least frequently reported that health providers asked whether support persons should be told about their condition or care (45.3%) (Supplement 1). Support persons most frequently reported that they felt welcome to ask health providers questions about women’s and newborns’ care (81.0%) and least frequently reported receiving information about the newborn’s care (17.1%).

The average PCMC score was 66.69 (SD 15.27) out of 100 with the Communication & Autonomy subscale scored lowest (mean 59.24, SD 21.34). Women generally rated satisfaction highly (mean 76.99, SD 18.580). Women reported an average of 17.84 (SD 5.03) key practices, representing 63.7% of practices assessed. On average, women reported 10.02 (SD 3.59) of 17 maternal key practices and 7.81 (SD 2.15) of 11 newborn key practices.

### Women’s PC-ISP experiences and quality of care

**Fig. 1:**
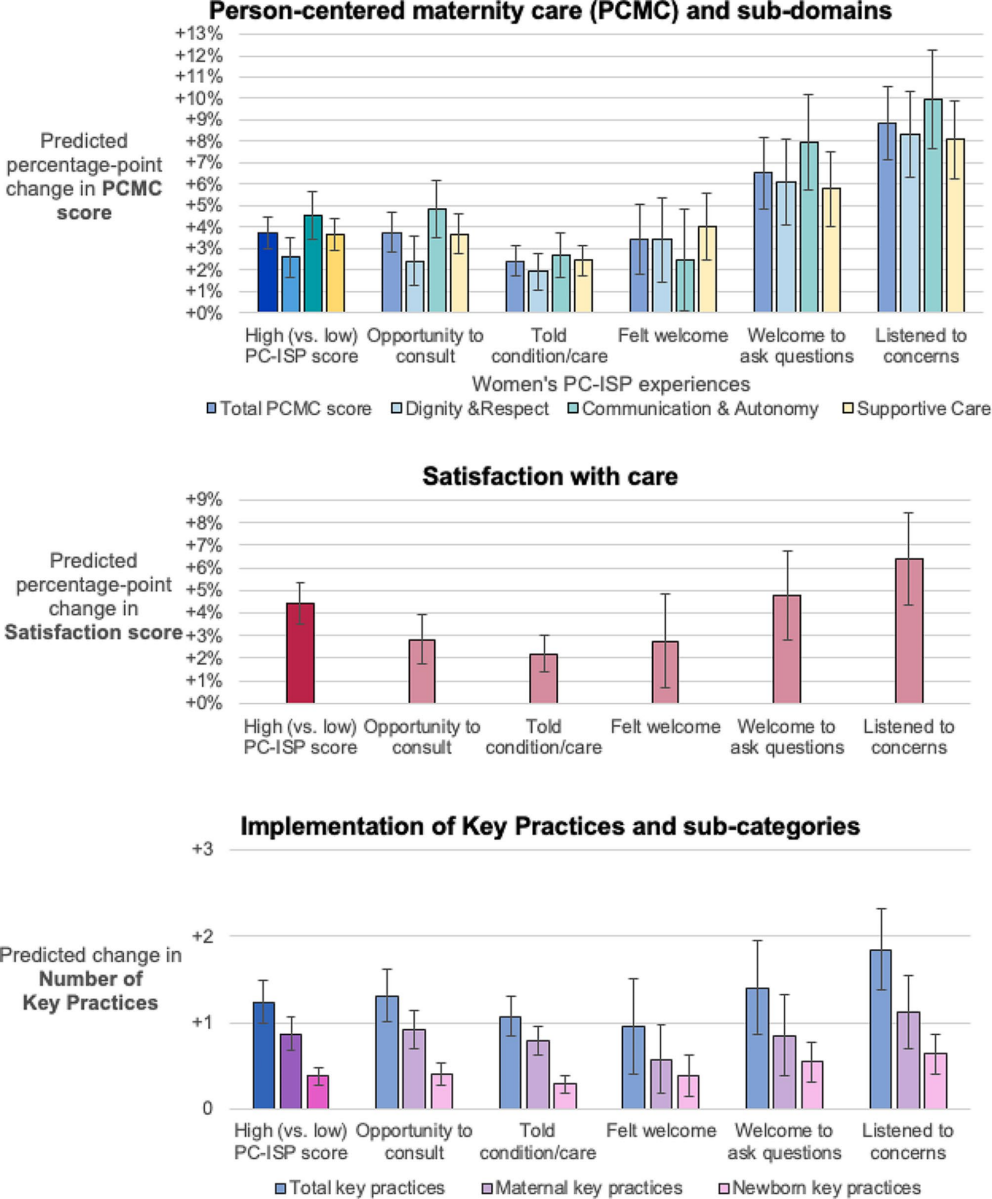
Associations of women’s PC-ISP measures and quality of care outcomes

In multivariable fractional regression models of women’s PC-ISP, high PC-ISP was associated with a 3.71%-point (95% CI: 2.95%, 4.46%) higher PCMC score compared to low PC-ISP (Table 3, models of individual indicators detailed in Supplement 2). Among individual indicators, women’s reports that providers *listened to concerns* were most strongly associated with total PCMC score (8.85%-point increase, 95% CI: 7.14%, 10.56%, Supplement 2). Of PCMC subdomains, high PC-ISP and most PC-ISP indicators (4 out of 5) were associated with the largest average increases in the *Communication & Autonomy* subdomain (Fig. 1). High PC-ISP also corresponded with an average 2.76%-point (95% CI: 1.86%, 3.65%) higher satisfaction score compared to low PC-ISP. All individual women’s PC-ISP indicators were associated with higher satisfaction and reports that providers *listened to concerns* were associated with the greatest increase in satisfaction (6.41%-point increase, 95% CI: 4.36%, 8.46%, Supplement 2).

For clinical outcomes, high (vs. low) PC-ISP was associated with a 4.49%-point (95% CI: 3.52%, 5.34%) increase in key clinical practices reported, corresponding to 1.24 (95% CI: 0.99, 1.50) more key clinical practices. Regarding types of clinical practices, women reporting high PC-ISP received 5.12%-point (95% CI: 4.01%, 6.22%) more maternal practices and 3.42%-point (95% CI: 2.43%, 4.41%) more newborn key practices than women reporting low PC-ISP. Providers *listening to support persons’ concerns* was associated with the greatest increase in key practices. All women’s individual PC-ISP indicators were associated with an increase in maternal and newborn key practices.

**Table 3:** Estimates of average marginal effects (percentage-point change) of women’s PC-ISP experiences and quality-of-care indicators from fractional regression results (*n* = 1,138)

	Independent variable: Women’s High (vs. low) PC-ISP (4 + vs. <4)		
	Unadj. dy/ex (95% CI)	Adjusted dy/ex.^1^ (95% CI)	
**Dependent variables**			
**Person-centered outcomes**			
Person-Centered Maternity Care (PCMC) (0-100)	3.91%*** (3.12%, 4.70%)	3.71%*** (2.95%, 4.46%)	
* Dignity & Respect subdomain (0-100)*	2.84%*** (1.92%, 3.77%)	2.59%*** (1.67%, 3.50%)	
* Communication & Autonomy subdomain (0-100)*	4.66%*** (3.50%, 5.81%)	4.55%*** (3.43%, 5.67%)	
* Supportive Care subdomain (0-100)*	3.90%*** (3.11%, 4.69%)	3.67%*** (2.92%, 4.41%)	
Satisfaction with care (0-100)	3.05%*** (2.13%, 3.97%)	2.76%*** (1.86%, 3.65%)	
**Clinical outcomes**			
Implementation of key clinical practices (0–28 practices)	4.49%*** (3.56%, 5.43%)	4.43%*** (3.52%, 5.34%)	
* Maternal key practices (0–17 practices)*	5.21%*** (4.08%, 6.33%)	5.12%** (4.01%, 6.22%)	
* Newborn key practices (0–11 practices)*	3.43%*** (2.42%, 4.44%)	3.42%*** (2.43%, 4.41%)	

*Note* *p < 0.05, **p < 0.01, ***p < 0.001

All coefficient estimates were calculated from separate models

^1^Adjusted coefficients’ models adjusted for *Women’s factors*: age, marital status, parity, educational attainment, current employment status, birthplace, household empowerment, health insurance coverage, self-rated health status; *Support Person factors*: support person type(s), total support persons, and timing of support; *Facility factors*: type of facility, total number of providers assisting delivery, whether women selected the facility because of quality, and if they were referred to the facility

### Support persons’ PC-ISP experiences and quality of care

No support persons’ indicators were associated with PCMC scores (Supplement 2). One support persons’ PC-ISP indicator was positively associated with higher satisfaction with care: support person’s experiences of being *provided information about the newborn* was associated with a 0.78%-point (95% CI: 0.25%, 1.31%) higher satisfaction score. In addition, support persons’ reports of being *provided information about the woman* and *information about the newborn* were both associated with increased key clinical practices: 0.99%-point (95% CI: 0.36%, 1.62%) and 0.59%-point (95% CI: 0.02%, 1.17%) increases in total key practices, respectively.

## Discussion

This study provides needed evidence that integrating support persons, as a critical part of respectful care, is associated with women’s positive experiences of person-centered care, higher satisfaction with care, and better clinical care experiences—supporting proposed links in the WHO QoC Framework [5]. These results also support literature suggesting that integrating support persons may lead to improved maternal and neonatal health outcomes [14]. The benefits of social support provided by doulas and support persons during labor and delivery are well-known (e.g., fewer cesarean deliveries, fewer neonatal intensive unit admissions) [6, 29], but most literature has proposed underlying psychosocial mechanisms, positing that support persons’ presence reduces the adverse consequences of fear and distress when women labor alone [6]. Our study highlights improved quality care as an additional mechanism as support persons can influence providers’ care delivery. Support persons are important advocates for women and newborns during care to garner respect from providers, call providers when medical attention is needed, and point out clinical gaps in care [25, 30]. Our findings support other recent studies that the presence of lay companions is associated with more respectful and person-centered care [8, 9], which may benefit maternal mental health and decrease complications [31]. 

Findings highlight very low access to intrapartum support and ways to better integrate support persons. Only 7% of women in the sample reported support persons during labor and delivery and one-in-five support persons received information about the woman or newborn. Health care systems need to improve communication between providers, women, and support persons, and facilitate support persons’ involvement with women’s and newborns’ care. Notably, integrating support persons was associated with beneficial increases in the Communication and Autonomy sub-domain, which is typically the lowest PCMC sub-domain in Kenya and elsewhere [32]. In addition, providers listening to support persons’ concerns was consistently the PC-ISP indicator most strongly associated with all outcomes investigated. Support persons can enhance communication between providers and women by expressing women’s concerns to providers and ensuring that providers’ directions are heard and understood. Furthermore, results suggest that support persons play an important role in newborn care [29] all women’s PC-ISP indicators were associated with increased newborn key practices. Support persons may aid families’ involvement in newborn care, allowing women to be aware of newborns’ well-being and care during their own postpartum care and recovery. This is particularly salient in this context where nearly one-in-five newborns were separated from mothers at the facility [33], underscoring women’s inability to be involved in newborn care.

Notably, our study found associations between women’s PC-ISP and care outcomes, but only found a few small associations between support persons’ PC-ISP and outcomes. These findings suggest that women’s perceptions of integrating support persons are more important for their experiences of care than support persons’ experiences of being integrated. It is plausible that this may highlight the importance of involving women in support person integration. Simply put, when support persons are integrated in ways that keep women at the center of their own care, they may perceive that care is of higher quality. However, it is also possible that the few PC-ISP measures were limited in their measurement of the many ways that support persons can be integrated. For example, we may not have captured all support persons’ experiences; we only surveyed one support person per woman and women often look to multiple individuals for different types of support. Additionally, PC-ISP measures asked about integrating family members but women also reported non-family support persons, so these measures may not adequately capture how non-family support persons were integrated. Finer-grained investigations are needed, using formally developed and more detailed measures of integrating support persons (e.g., content and nature of communication between women, support persons, and providers).

The self-reported outcome measures have some drawbacks as well as some advantages. For example, satisfaction ratings rely on perceived quality, which can be a crude measure that is subject to women’s low expectations and knowledge of standards. The key practices measure may also be prone to recall error especially if women were not aware of or remember all the procedures conducted. Our study is unique in that we used three different self-reported measures of quality care, to comprehensively assess multiple aspects of quality care from women’s own perspectives. One advantage to our approach is that we assessed quality care based on measures of specific practices and procedures rather than perceived quality alone. In addition, evidence indicates that women can accurately report on multiple aspects of care for themselves and their newborns [34]. 

Lastly, our use of cross-sectional surveys limits our ability to draw causal conclusions and reverse causality is possible: women with better experiences of care may report that their support persons were more integrated. Results may be generalizable to other urban facilities in Kenya, but generalizability may be limited since this study was conducted in a small number of facilities in urban Nairobi and Kiambu counties. Future studies should further explore how the local and facility context modifies the relationships between integrating support persons and quality care, especially since the facility context critically determines women’s access to support persons [11]. Additionally, within this context, our study did not include any support persons who were trained or experienced support persons (i.e., doulas, TBAs, etc.) so our findings may not be generalizable to contexts where professional support persons are common. However, because professional support persons can improve safety for women and newborns yet still face barriers to inclusion in maternity care [6, 14], future research should evaluate quality of care and its relationship to integrating professional support persons.

## Conclusions

Health care systems and providers should regard support persons as assets and explore ways that women, support persons, providers, and facilities can cooperatively deliver and monitor maternity care. In Kenya, models of care that have encouraged collaborations, such as those between health centres and communities, have demonstrated ability to leverage their strengths to improve obstetric outcomes and increase respectful maternity care [35]. Integrating support persons as a part of respectful care is a key strategy to unify women, communities, and providers to improve maternal and newborn health.

### Electronic supplementary material

Below is the link to the electronic supplementary material.

Supplementary Material 1

Supplementary Material 2

## Data Availability

Data that support the findings of this study are available from the corresponding author upon reasonable request.

## Abbreviations

PC-ISP: Person-Centered Integration of Support Persons
PCMC: Person-Centered Maternity Care
QoC: Quality of Care
WHO: World Health Organization
