# Supplementary material for: Integrating support persons into maternity care and associations with quality of care: a postpartum survey of mothers and support persons in Kenya
Source: BMC Pregnancy Childbirth. 2024 Jun 13;24:425. doi: 10.1186/s12884-024-06611-y (PMC11170830; doi:10.1186/s12884-024-06611-y)
Supplement: Supplementary file 1 — Supplementary Material 1 [file 12884_2024_6611_MOESM1_ESM.docx]

**Supplement Table 1. Distributions of key variables: Women’s and SPs’ experiences of PC-ISP and Quality of care outcomes**

| **Women’s PC-ISP Experiences** | | | | | | | | |  |
| --- | --- | --- | --- | --- | --- | --- | --- | --- | --- |
| **Sub-construct** | | | **Variable name** | | | **“Agree/**  **somewhat agree”**  **N** | | **%** | |
| Decision-making support | | | *Opportunity to consult* | | | 670 | | 58.9% | |
| Communication and provision of information | | | *Told condition/care* | | | 516 | | 45.3% | |
| Welcoming environment | | | *Felt welcome* | | | 896 | | 78.7% | |
| Ability to ask questions and express concerns | | | *Welcome to ask questions ^a^* | | | 903 | | 80.7% | |
|  |  |  | *Listened to concerns ^b^* | | | 912 | | 81.9% | |
|  |  |  | | **N** | | | **Mean (SD) or %** | |  |
| Summative Women’s PC-ISP score (range 0-5) | | | | | 1,138 | | 3.42 (1.36) | |  |
| High PC-ISP (4 or more) | | | | | 583 | | 51.2% | |  |
| Low PC-ISP (3 or less) | | | | | 555 | | 48.8% | |  |
| **Support persons’ PC-ISP Experiences** | | | | | | | | |  |
| **Sub-construct** | | | **Variable name** | | | **“Yes”**  **N** | | **%** | |
| Communication and provision of information | | | *Provided info about woman ^c^* | | | 119 | | 19.7% | |
|  |  |  | *Provided info about newborn ^d^* | | | 103 | | 17.1% | |
| Ability to ask questions and express concerns | | | *Welcome to ask questions* | | | 490 | | 81.0% | |
|  |  |  | | **N** | | | **Mean (SD)** | |  |
| Summative Support Persons’ PC-ISP score (range 0-3) | | | | | 606 | | 1.17 (0.87) | |  |
| **Quality of care outcomes** | | | | | **N** | | **Mean (SD)** | |  |
| Person-Centered Maternity Care Scale (PCMC) scale total (range 0-100) ^e^ | | | | | 1,138 | | 66.69 (15.27) | |  |
| *Dignity & Respect (0-100)* | | | | | 1,138 | | 77.34 (18.46) | |  |
| *Communication & Autonomy (0-100)* | | | | | 1,138 | | 59.24 (21.34) | |  |
| *Supportive Care (0-100)* | | | | | 1,138 | | 66.90 (15.16) | |  |
| Satisfaction score total (0-100) | | | | | 1,138 | | 76.99 (18.58) | |  |
| Implementation of key practices (0-28 practices) | | | | | 1,138 | | 17.84 (5.03) | |  |
| *Maternal key practices (0-17 practices)* | | | | | 1,138 | | 10.02 (3.59) | |  |
| *Newborn key practices (0-11 practices)* | | | | | 1,138 | | 7.81 (2.15) | |  |

^a^ 19 women responded N/A to this question

^b^ 24 women responded N/A to this question

^c^ One refused to answer this question

^d^ One refused to answer and one missing response for this question

^e^ PCMC and all sub-scales were standardized to a 100-point scale
