# Supplementary material for: Integrating support persons into maternity care and associations with quality of care: a postpartum survey of mothers and support persons in Kenya
Source: BMC Pregnancy Childbirth. 2024 Jun 13;24:425. doi: 10.1186/s12884-024-06611-y (PMC11170830; doi:10.1186/s12884-024-06611-y)
Supplement: Supplementary file 2 — Supplementary Material 2 [file 12884_2024_6611_MOESM2_ESM.docx]

**Supplement 2. Estimated average marginal effects (percentage-point change) of women’s and SPs’ PC-ISP experiences on quality of care outcomes from fractional regression results**

|  | **PCMC total score** | | **PCMC Sub-domains** | | | **Satisfaction with care** | **Total Key Practices** | **Key Practice**  **Sub-categories** | |  |
| --- | --- | --- | --- | --- | --- | --- | --- | --- | --- | --- |
|  | **N** | dy/ex  (95% CI) | **Dignity & Respect**  dy/ex  (95% CI) | **Communication**  **& Autonomy**  dy/ex  (95% CI) | **Supportive Care**  dy/ex  (95% CI) | dy/ex  (95% CI) | dy/ex  (95% CI) | **Maternal Practices**  dy/ex  (95% CI) | **Newborn Practices**  dy/ex  (95% CI) |  |
| **Women’s PC-ISP experiences** | | |  |  |  |  |  |  |  |  |
| Opportunity to consult | 1,138 | 3.76%***  (2.84%, 4.68%) | 2.42%***  (1.28%, 3.55%) | 4.84%***  (3.51%, 6.17%) | 3.68%***  (2.76%, 4.60%) | 2.84%***  (1.75%, 3.92%) | 4.68%***  (3.58%, 5.77%) | 5.39%***  (4.08%, 6.69%) | 3.64%***  (2.44%, 4.83%) | |
| Told condition | 1,138 | 2.40%***  (1.70%, 3.11%) | 1.93%***  (1.09%, 2.76%) | 2.66%***  (1.63%, 3.70%) | 2.44%***  (1.75%, 3.13%) | 2.19%***  (1.36%, 3.01%) | 3.83%***  (3.02%, 4.65%) | 4.66%***  (3.68%, 5.64%) | 2.61%**  (1.70%, 3.52%) | |
| Felt welcome | 1,138 | 3.44%***  (1.79%, 5.09%) | 3.40%**  (1.42%, 5.37%) | 2.46%*  (0.12%, 4.81%) | 4.02%***  (2.43%, 5.61%) | 2.76%**  (0.71%, 4.82%) | 3.42%**  (1.43%, 5.40%) | 3.39%**  (1.05%, 5.73%) | 3.46%**  (1.32%, 5.60%) | |
| Welcome to ask questions ^1^ | 1,119 | 6.52%***  (4.87%, 8.16%) | 6.10%***  (4.12%, 8.09%) | 7.97%***  (5.75%, 10.19%) | 5.79%***  (4.05%, 7.52%) | 4.78%***  (2.80%, 6.76%) | 5.00%***  (3.04%, 6.94%) | 5.01%***  (2.66%, 7.79%) | 4.95%***  (2.81, 7.09%) | |
| Listened to concerns ^2^ | 1,114 | 8.85%***  (7.14%, 10.56%) | 8.31%***  (6.32%, 10.30%) | 9.95%***  (7.65%, 12.25%) | 8.07%***  (6.28%, 9.86%) | 6.41%***  (4.36%, 8.46%) | 6.27%***  (4.26%, 8.28%) | 6.59%***  (4.14%, 9.04%) | 5.76%***  (3.60%, 7.92%) | |
| **Support persons’ PC-ISP experiences** | | |  |  |  |  |  |  |  |  |
| Provided info about woman ^3^ | 605 | 0.44%  (-0.10%, 0.98%) | — | — | — | 0.37%  (-0.27%, 1.02%) | 0.99%**  (0.36%, 1.62%) | — | — |  |
| Provided info about newborn ^4^ | 604 | 0.40%  (-0.07%, 0.88%) | — | — | — | 0.78%*  (0.25%, 1.31%) | 0.59%*  (0.02%, 1.17%) | — | — |  |
| Welcome to ask questions ^3^ | 605 | 1.73%  (-0.93%, 4.39%) | — | — | — | 2.14%  (-0.65%, 4.92%) | 1.41%  (-1.39%, 4.22%) | — | — |  |

Notes: *p<0.05, **p<0.01, ***p<0.001

^1^ 19 women responded N/A to this question

^2^ 24 women responded N/A to this question

^3^ One refused to answer for this question

^4^ One refused to answer and one missing response for this question

All models adjusted for *Women’s factors*: age, marital status, parity, educational attainment, current employment status, birthplace, household empowerment, health insurance coverage, self-rated health status; *Support Person factors:* support person type(s), total support persons, and timing of support; *Facility factors:* type of facility, total number of providers assisting delivery, whether women selected the facility because of quality, and if they were referred to the facility.
